# ChR2 Mutants at L132 and T159 with Improved Operational Light Sensitivity for Vision Restoration

**DOI:** 10.1371/journal.pone.0098924

**Published:** 2014-06-05

**Authors:** Zhuo-Hua Pan, Tushar H. Ganjawala, Qi Lu, Elena Ivanova, Zhifei Zhang

**Affiliations:** 1 Department of Anatomy/Cell Biology, Wayne State University School of Medicine, Detroit, Michigan, United States of America; 2 Department of Ophthalmology, Kresge Eye Institute, Wayne State University School of Medicine, Detroit, Michigan, United States of America; Oregon Health & Science University, United States of America

## Abstract

The ectopic expression of microbial opsin-based optogenetic sensors, such as channelrhodopsin-2 (ChR2) in surviving inner retinal neurons, is a promising approach to restoring vision after retinal degeneration. However, a major limitation in using native ChR2 as a light sensor for vision restoration is the low light sensitivity of its expressing cells. Recently, two ChR2 mutations, T159C and L132C, were reported to produce higher photocurrents or have ultra light sensitivity. In this study, we created additional ChR2 mutants at these two sites to search for more light responsive ChR2 forms and evaluate their suitability for vision restoration by examining their light responsive properties in HEK cells and mouse retinal ganglion cells. We found additional ChR2 mutants at these two sites that showed a further increase in current amplitude at low light levels in the cells expressing these mutants, or operational light sensitivity. However, the increase in the operational light sensitivity was correlated with a decrease in temporal kinetics. Therefore, there is a trade-off between operational light sensitivity and temporal resolution for these more light responsive ChR2 mutants. Our results showed that for the two most light responsive mutants, L132C/T159C and L132C/T159S, the required light intensities for generating the threshold spiking activity in retinal ganglion cells were 1.5 and nearly 2 log units lower than wild-type ChR2 (wt-ChR2), respectively. Additionally, their ChR2-mediated spiking activities could follow flicker frequencies up to 20 and 10 Hz, respectively, at light intensities up to 1.5 log units above their threshold levels. Thus, the use of these more light responsive ChR2 mutants could make the optogenetic approach to restoring vision more feasible.

## Introduction

The ectopic expression of light sensors in surviving retinal neurons, including retinal ganglion cells (RGCs), to impart retinal light sensitivity has been a promising approach to restoring vision after retinal degeneration [1]–[12]. Channelrhodopsin (ChR)-based microbial opsins, such as channelrhodopsin-2 (ChR2), are the most versatile and powerful optogenetic sensors because they are genetically encoded light-gated channels, and their biophysical properties can be altered by genetic engineering [13]–[15]. However, a major limitation in the use of native ChR2 as a light sensor for vision restoration is the low light sensitivity of its expressing cells [1]. The light sensitivity of ChR2-expressing RGCs is at least 4 log units lower than the sensitivity of cone photoreceptors. To improve the light sensitivity of ChR2-expressing RGCs requires developing ChR2 variants that can produce large photocurrents at low light intensities, also referred to as operational light sensitivity [15]. Although light-intensifying devices could be used to expand the operational light range of ChR2, the development of ChR2 or ChR with high operational light sensitivity can avoid the need to use extremely bright light that could potentially cause phototoxicity or other complications.

Since the discovery of native ChR1 and ChR2 [13], [14], many ChR2 mutants and ChR variants with altered light response properties, including increased photocurrents, have been reported [15]–[22]. In particular, the T159C and L132C ChR2 mutations have been reported to produce in larger photocurrents or an ultra light-sensitive ChR2 [20], [21]. More recently, a further increase in photocurrents was reported for the L132C/T159C mutant [23]. Thus, it would be interesting to investigate whether other mutations at these two sites could result in higher operational light sensitivity.

The increased photocurrents of the L132C mutant were reported to be mediated by increased Ca^2+^ permeability [21]. However, other studies have shown that in general, increased photocurrents in ChR mutants, including the L132C mutant, are correlated to their prolonged deactivation kinetics or off rates [15], [23]. The increase in the deactivation kinetics would be expected to affect the temporal resolution of the signals that can be encoded by ChR2. Thus, evaluating these ChR2 mutants in retinal neurons is important to assess their suitability in vision restoration.

To search for more light responsive ChR2 mutants and evaluate their suitability for vision restoration, we created additional ChR2 mutants at these two sites. We examined their light response properties and compared them with the previously reported mutants in HEK cells and RGCs. We identified additional mutations at these two sites that produced further increases in operational light sensitivity. Although the increase in operational light sensitivity for these mutants was correlated with a decrease in temporal coding abilities, the use of these ChR2 mutants could make the optogenetic approach to restoring vision more feasible.

## Materials and Methods

### DNA and Viral Vector Constructs

The single or double ChR2 mutations at the L132 and T159 sites were created by gene synthesis (GenScript, Piscataway, NJ, USA) or site-directed mutagenesis (Agilent Technologies, Santa Clara, CA, USA). rAAV2 vectors carrying a fusion construct of ChR2 and GFP (ChR2-GFP) and driven by a CAG (a hybrid CMV early enhancer/chicken β-actin) promoter were modified from a previously reported construct [1]. The virus vectors were packaged at the Gene Therapy Program of the University of Pennsylvania or Virovek (Hayward, CA, USA).

### Animals and Viral Vector Injection

All animal experiments and procedures were approved by the Institutional Animal Care and Use Committee of Wayne State University and were performed in accordance with the NIH *Guide for the Care and Use of Laboratory Animals*. Intravitreal viral injections were performed on adult C57BL/6J mice between 1 and 2 months of age according to previously described procedures [1]. The concentrations of the viral vectors were 4.7–8.0×10^12 ^GC/ml. One to two months after viral injection, animals were euthanized by CO_2_ asphyxiation followed by decapitation for electrophysiological recordings.

Enucleated eyes were fixed in 4% paraformaldehyde in phosphate buffer (PB) at room temperature for 20 minutes. Fluorescence expression was examined in flat-mounted retinas. All images were acquired using a Zeiss Axioplan 2 microscope with Apotome (Carl Zeiss, Oberkochen, Germany) with the AxioVision software.

### HEK Cell Culture, DNA Transfection, Fluorescence Measurements, and Patch-clamp Recordings

HEK-293F cells were maintained in Advanced Dulbecco’s Minimum Essential Medium (Life Technologies, Grand Island, NY, USA) supplemented with 5% fetal bovine serum, MEM Non-Essential Amino Acid Solution (100×), 100 units/ml penicillin G and 100 µg/mL streptomycin at 37°C in a humidified 5% CO_2_ atmosphere. In preparation for DNA transfection, the cells were seeded in 35-mm dishes and then transfected with rAAV2 DNA vectors carrying wt-ChR2 or mutant ChR2s using Lipofectamine 2000 (Life Technologies). *All*-trans-retinal (1 µM) was added to the culture media at the time of DNA transfection. Fluorescence measurements and patch-clamp recordings were performed 2 days after DNA transfection.

For fluorescence measurements, HEK cells were fixed with 4% paraformaldehyde in 0.1 M phosphate buffer (PB) for 10 min and then washed in 0.1 M PB 3 times for 10 min each. All images were captured under fixed conditions (light intensity and exposure time) using a Zeiss Axioplan 2 microscope (Carl Zeiss) with the Apotome oscillating grating to reduce out-of-focus stray light. The fluorescence intensity of each HEK cell was calculated from a single optical section by dividing the total fluorescence value of its plasma membrane region by the total number of pixels of the same region using the ImageJ software, as previously described [18].

Recordings with patch electrodes in the whole-cell configuration were acquired using standard procedures at room temperature (20–25°C) with an EPC-9 amplifier and PULSE software (Heka Electronik, Lambrecht/Pfalz, Germany). The electrodes were coated with a silicone elastomer (Dow Corning, Midland, MI, USA) and fire-polished. The resistance of the electrode was 5–8 MΩ. The series resistance ranged from 8 to 15 MΩ. Cell capacitance was cancelled using the PULSE software. Recordings were performed in Hank’s balanced salt solution containing (in mM): 138 NaCl, 1 NaHCO_3_, 0.3 Na_2_HPO_4_, 5 KCl, 0.3 KH_2_PO_4_, 1.25 CaCl_2_, 0.5 MgSO_4_, 0.5 MgCl_2_, 5 HEPES, 22.2 glucose, and 0.001% (v/v) phenol red, and the pH was adjusted to 7.2 using 0.3 N NaOH. *All*-trans-retinal (1 µM) was added to the recording solution. The electrode solution contained (in mM): 110 Cs-Methanesulfonate, 30 TEA-Cl, 2 MgCl_2_, 0.1 CaCl_2_, 10 EGTA, and 10 HEPES. The pH was adjusted to 7.25 using CsOH. Liquid junction potentials were measured and corrected.

### Multielectrode Array Recordings and Data Analysis

The multielectrode array recordings were performed as previously described [1], [24]. Briefly, the retina was dissected and placed photoreceptor side down on a piece of nitrocellulose filter paper (Millipore Corp., Bedford, MA, USA). The mounted retina was placed in the MEA-60 multielectrode array recording chamber with 30-µm diameter electrodes spaced 200 µm apart (Multi Channel System MCS GmbH, Reutlingen, Germany). The ganglion cell layer was facing the recording electrodes. The retina was continuously perfused with an oxygenated extracellular solution at 34°C during all experiments. The extracellular solution contained (in mM): 124 NaCl, 2.5 KCl, 2 CaCl_2_, 2 MgCl_2_, 1.25 NaH_2_PO_4_, 26 NaHCO_3_, and 22 glucose at a pH of 7.35 with 95% O_2_ and 5% CO_2_. Recordings usually began 60 min after the retina was positioned in the recording chamber. Light stimuli were projected onto the ganglion cell side of the retina. The interval between the on-set of each light stimulus was 20 s. Signals were filtered between 200 Hz (low cutoff) and 20 kHz (high cutoff). A threshold of 17–34 µV was used to detect action potentials, and action potentials from individual neurons were determined via a standard expectation-maximization algorithm with the off-line Sorter software (Plexon, Inc., Dallas, TX, USA). Spike raster plots and averaged spike rate histograms were generated using the NeuroExplorer software (Nex Technologies, Madison, AL, USA).

The relationship between the response-amplitude attenuation and frequency was measured from the averaged spike rate histograms. For each frequency, the attenuation value was calculated by dividing the average trough-to-crest amplitude by the average peak amplitude from the second five-responses to pulse stimuli. The attenuation values were converted to a decibel (dB) scale.

### Light Stimulation

Light stimuli were generated by a 150 W xenon lamp-based scanning monochromator at a wavelength of 460 nm and a bandwidth of 10 nm (TILL Photonics, Munich, Germany). In the HEK cell recordings, light stimuli were coupled to the microscope with an optical fiber. In multielectrode recordings, the light stimuli were directly projected onto the bottom of the recording chamber through an optical fiber. The light intensity was adjusted using neutral density filters.

### Chemicals

D(−)-2-Amino-5-phosphonopentanoic acid (D-AP5), L-(+)-2-Amino-4-phosphonobutyric acid (L-APB), and 6-Cyano-7-nitroquinoxaline-2,3-dione (CNQX) were purchased from Sigma-Aldrich (St. Louis, MO, USA).

## Results

### Comparison of Photocurrent Amplitudes and Temporal Kinetics of ChR2 Mutants in HEK Cells

**Figure 1:**
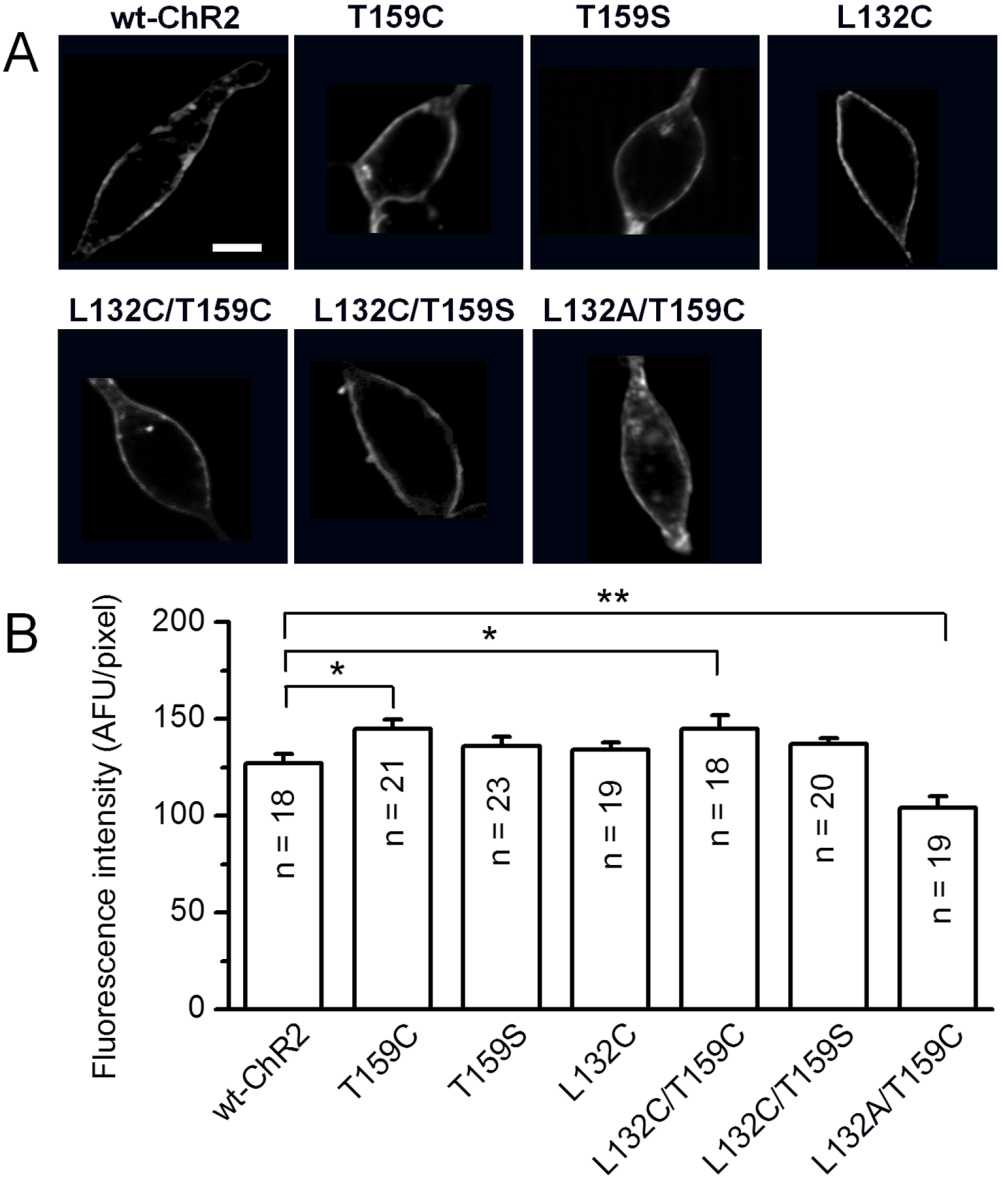
Comparison of membrane expression and fluorescence intensities in HEK cells. A) Representative images showing the expression of ChR2-GFP for wt-ChR2, T159C, T159S, L132C, L132C/T159C, L132C/T159S, and L132A/T159C. Scale bar, 10 µm. B) Average fluorescent intensities measured in the plasma membrane region. The data are shown in arbitrary fluorescent units (AFU) as the mean **±** SEM from the indicated number of cells. *p<0.05, **p<0.01.

To examine how mutations at L132 and/or T159 affect ChR2-mediated light response properties and to search for more light responsive ChR2s, we created a number of mutants by replacing these two amino acids with A, S, C, V, G, K, or D individually and in combination, including the previously reported L132C and T159C mutants [20], [21] and the more recently reported dual mutation of L132C/T159C [23]. Their expression and light response properties were first examined in HEK cells. Six mutants (T159S, L132C/T159S, and L132A/T159C along with three previously reported mutants, L132C, T159C, and L132C/T159C) had satisfactory expression in HEK cells based on GFP expression with the exception of L132A/T159C, which exhibited some intracellular aggregation. Representative fluorescence images of these six mutants along with wt-ChR2 are shown in Fig. 1A, and the fluorescence intensities measured in the plasma membrane region are shown in Fig. 1B. The fluorescence intensities of the L159C and L132C/T159C mutants were 14% higher, but the intensity of the L132A/T159C mutant was 18% lower than wt-ChR2. These six mutants had large light-evoked currents (Table 1). Other mutants had various expression problems. Four mutants (L132A, L132S, L132A/T159A, and L132S/T159A) showed poor expression (data not shown) and exhibited smaller ChR2-mediated currents (Table 1). The remaining mutants showed very poor or no GFP expression and their light responses were not examined.

**Table 1:** The properties of light-elicited currents of wt-ChR2 and ChR2 mutants in HEK cells.

mutation	I_peak_ (pA)	I_plateau_ (pA)	t_time to peak_ (ms)	τ_off_ (ms)	I_peak_ (pA)	τ_on_ (ms)	τ_off_ (ms)	n
	ND = 0	ND = 0	ND = 0	ND = 0	ND = 2.5	ND = 2	ND = 2.5	
WT	782±84	250±36	15.1±1.8	18.2±1.0	25.7±2.5	20.5±2.6	27±1.7	7
L132A	400±52	318±44		734±71				4
L132S	318±73	221±54		310±30				4
L132C	1268±47	950±36	25.8±0.61	90±4.6	125±9.8	52±3.0	65±4.2	8
T159S	1349±73	459±42	25.9±0.80	130±15	116±19	72±3.1	77±1.8	6
T159C	1239±82	407±26	17.6±0.53	45±2.9	62±5.5	33±1.4	54±3.8	6
L132A/T159A	452±160			10.4±2.3×10^3^				4
L132S/T159A	299±67			8.2±0.8×10^3^				4
L132A/T159C	784±46	661±42	37.0±1.7	1390±63	399±22	439±31	1128±50	6
L132C/T159S	1037±69	993±65	45.6±1.5	1090±64	470±34	357±21	905±57	9
L132C/T159C	1062±11	830±85	30.3±1.1	199±17	212±19	127±11	175±7.1	6

The peak and plateau currents for all mutants, except L132A/T59S and L132S/T159A, were elicited by a 1-s light pulse. The peak currents for the L132A/T59S and L132S/T159A mutants were elicited by a 10-ms light pulse. The τ_on_ and τ_off_ constants were obtained by fitting a single exponential function to the decaying phase of the currents. The light intensities are presented with neutral density (ND) attenuation. The light intensity without (ND = 0) is 1.2×10^18^ photons/cm^2^s. The data are presented as the mean **±** SEM.

**Figure 2:**
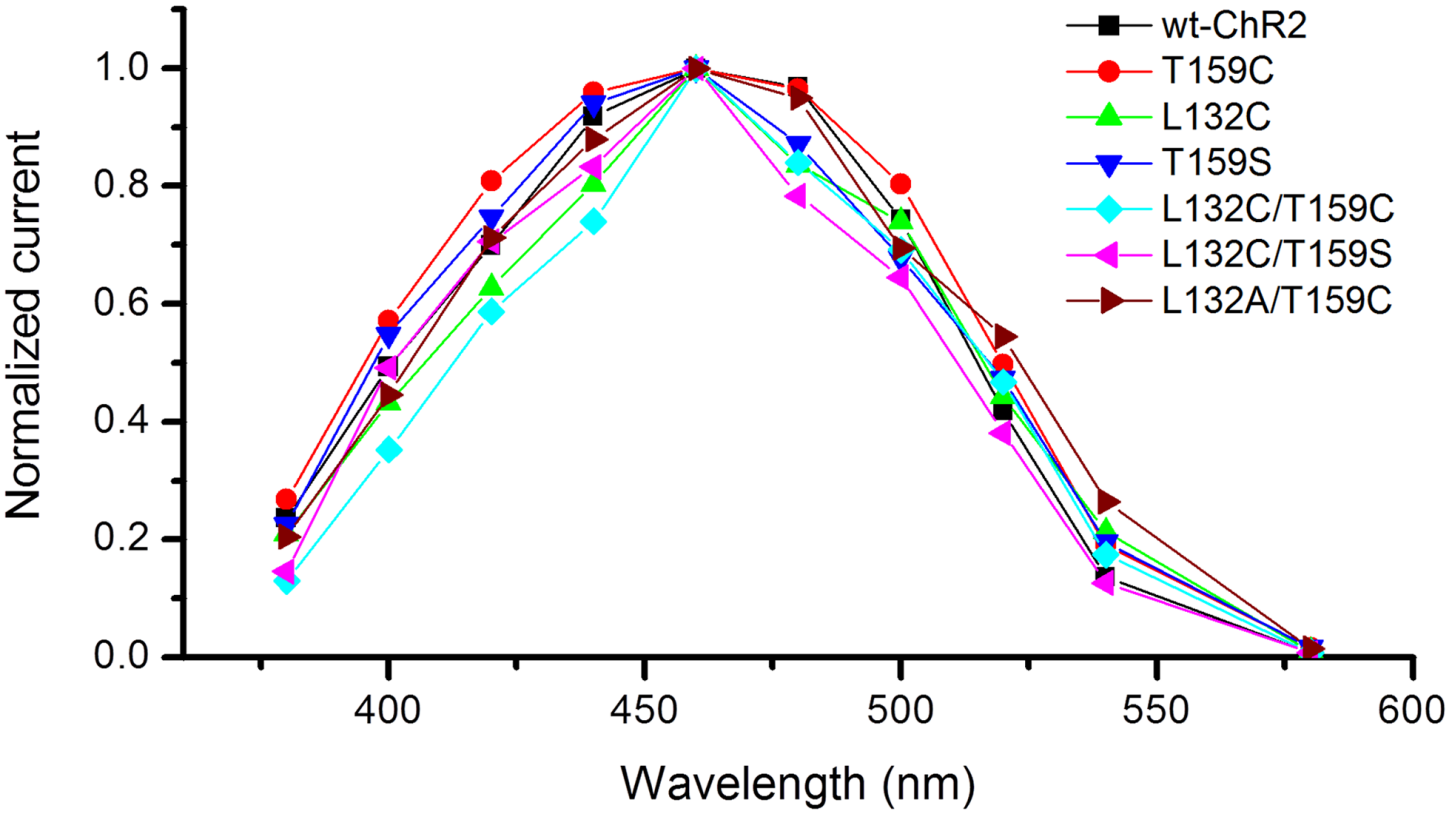
Comparison of the spectral response properties of the wt-ChR2 and ChR2 mutants in HEK cells. Representative spectral response curves for wt-ChR2, T159C, L132C, T159S, L132C/T159C, L132C/T159S, and L132A/T159C. Recordings were acquired in voltage-clamp with the membrane potential held at −60 mV. The current traces were evoked by 300-ms light pulses at different light wavelengths with a ND = 0 for wt-ChR2, T159C, and T159S, and with ND = 1 for L132C, L132C/T159C, L132C/T159S, and L132A/T159C. The measured light intensities (photons/cm^2^s) at different wavelengths with a ND = 0 were: 0.28×10^18^ (380 nm); 0.50×10^18^ (400 nm); 0.73×10^18^ (420 nm); 0.90×10^18^ (440 nm); 1.2×10^18^ (460 nm); 1.4×10^18^ (480 nm); 1.4×10^18^ (500 nm); 1.3×10^18^ (520 nm); 1.3×10^18^ (540 nm); and 1.3×10^18^ (580 nm). The measured light intensities at different wavelengths with a ND = 1 were: 0.46×10^17^ (380 nm); 0.77×10^17^ (400 nm); 1.1×10^17^ (420 nm); 1.4×10^17^ (440 nm); 1.7×10^17^ (460 nm); 1.9×10^17^ (480 nm); 1.9×10^17^ (500 nm); 1.8×10^17^ (520 nm); 1.7×10^17^ (540 nm); and 1.5×10^17^ (580 nm).

**Figure 3:**
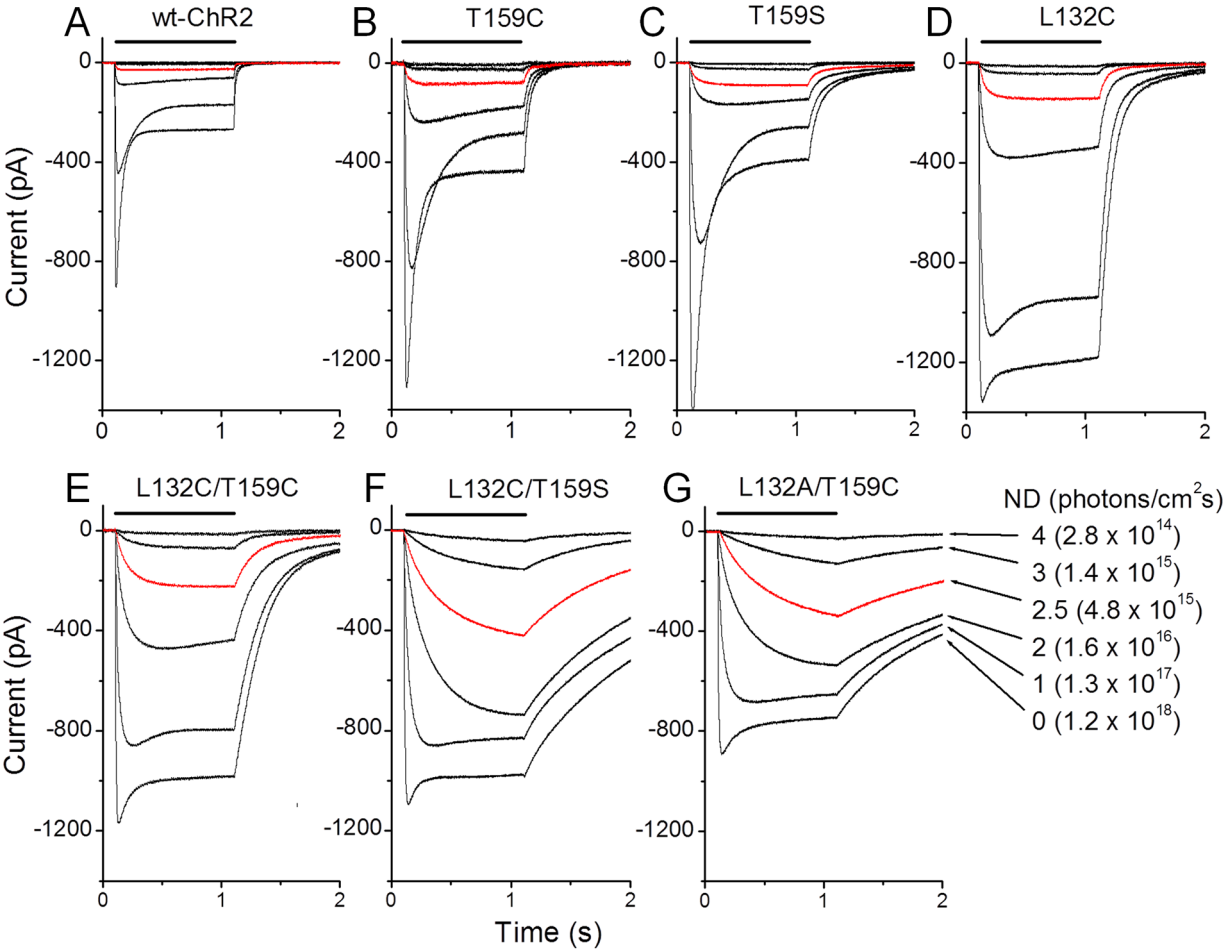
ChR2-mediated currents for wt-ChR2 and six ChR2 mutants in HEK cells. A–G) ChR2-mediated currents from wt-ChR2 (A), T159C (B), T159S (C), L132C (D), L132C/T159C (E), L132C/T159S (F), and L132A/T159C (G). Recordings were acquired in voltage-clamp with the membrane potential held at −60 mV. The current traces were evoked by 1-s light pulses at 460 nm with incremental light intensities attenuated by NDs of 4, 3, 2.5, 2, 1, and 0. The light intensities were measured as photons/cm^2 ^s. The red traces were elicited by light with a ND of 2.5.

The light response properties of the six mutants that exhibited good or relatively good expression were examined and compared with wt-ChR2. The spectral properties of these mutants were found to be unaltered (Fig. 2; also see [20], [21]). Therefore, all of the experiments that aimed to characterize their detailed light response properties were performed using 460 nm light, which is near the peak sensitive wavelength of wt-ChR2 [14]. Fig. 3A–G shows the representative current traces evoked by a 1-s light pulse with incremental light intensities (attenuated by the neutral density [ND] filters 4, 3, 2.5, 2, 1, and 0). After stimulating with the brightest light intensity (ND = 0), all of these mutants, except L132A/T159C, exhibited a much larger peak current than wt-ChR2. The desensitization of most mutants, except T159C and T159S, was largely abolished (Fig. 3D–G) and resulted in much larger plateau currents compared with wt-ChR2.

**Figure 4:**
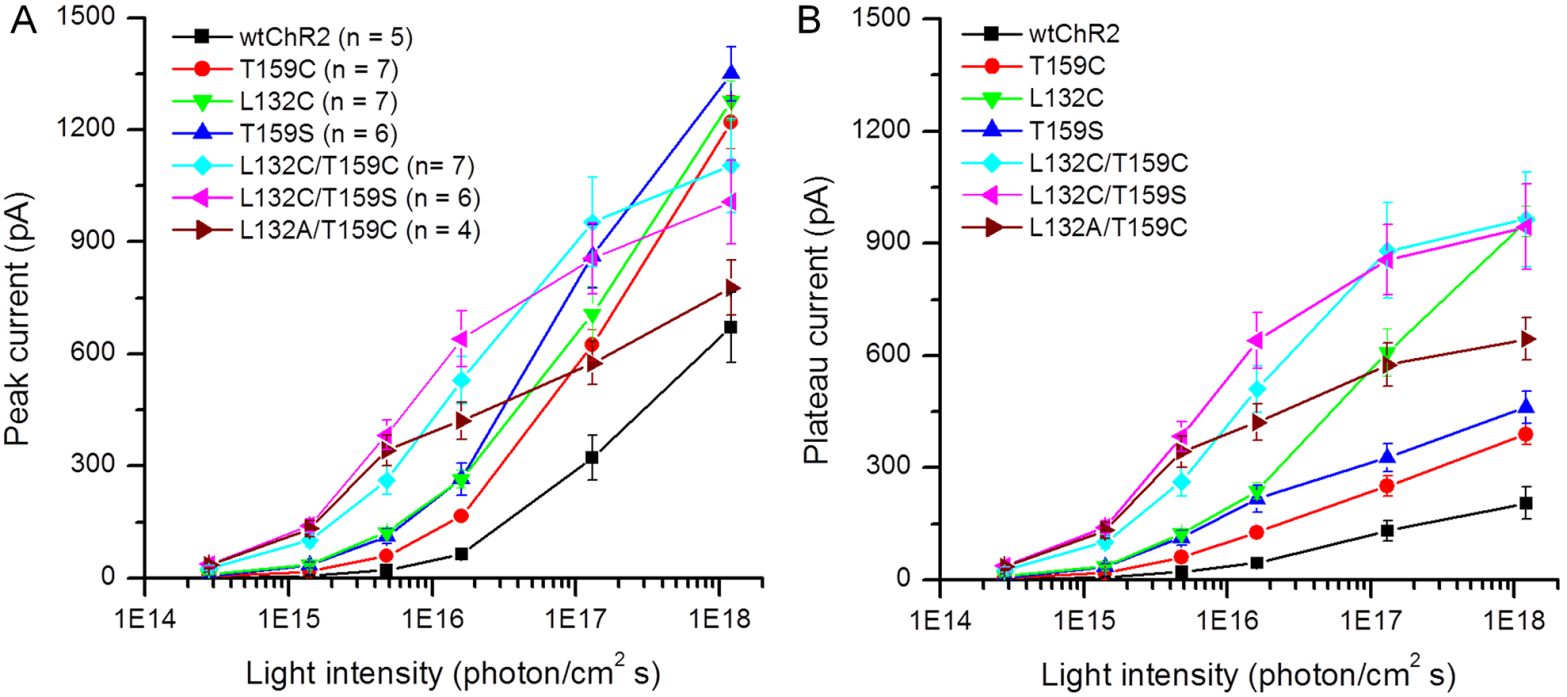
The light intensities and response relationships for the ChR2-mediated currents of wt-ChR2 and six ChR2 mutants in HEK cells. A, B) The light intensity response curves for the peak (A) and plateau currents (B) for wt and six mutant ChR2. The data are shown as the mean ± SEM.

The light intensity response curves for peak and plateau currents (measured at the end of the light pulse) are shown in Fig. 4A and B, respectively. All six mutants showed an increase in operational light sensitivity, as evidenced by the increased current amplitudes to light stimuli at low intensities. The increase in photocurrents to low light intensities was greater for the three double mutants, L132C/T159C, L132C/T159S, and L132A/T159C. This result could be more clearly visualized when the light-evoked currents were compared at a light intensity with a 2.5 ND attenuation (see red traces in Fig. 3).

**Figure 5:**
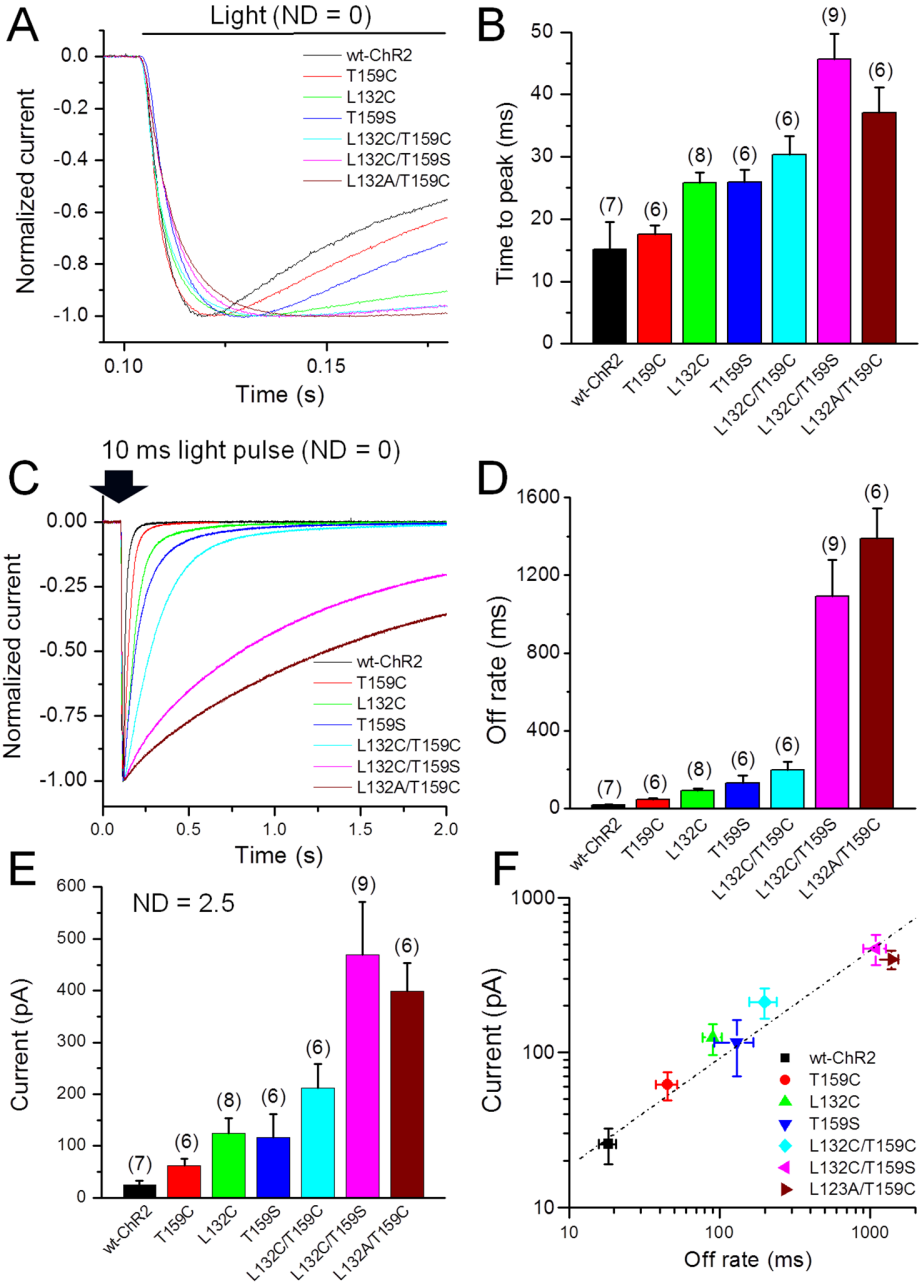
Comparison of the kinetics of the wt- and mutant ChR2-mediated currents and the relationship between current amplitude and deactivation in HEK cells. A) Representative current traces elicited by a 1-s light pulse with a ND = 0 to compare activation kinetics. B) The average values of the time to peak current. C) Representative traces elicited by a 10-ms light pulse with a ND = 0 for to compare deactivation kinetics. D) The average values of off rates or deactivation time constants. The deactivation time constants were obtained by fitting a single exponential function to the decaying phase of currents elicited by a 10-ms light pulse with a ND = 0. E) The average peak currents elicited by low light-intensity with a ND = 2.5. F) The plot of the currents elicited by light with a ND = 2.5, and the off rates are shown in (D). The data are presented as the mean ± SD.

The activation (on rate) and deactivation (off rate) kinetics of these more light responsive mutants were all slower than the kinetics of wt-ChR2. We quantitatively compared the activation kinetics by measuring the time to peak current evoked by a 1-s light pulse at a ND = 0 (Fig. 5A) and the on rate by fitting the rising phase of the currents elicited by light at a ND = 2.5. All of these mutants displayed an increase in the time to peak current (Fig. 5B) and the on rate compared with wt-ChR2 (see Table 1). The increase in the on rate of the currents elicited by the low light stimulation (ND = 2.5) was particularly marked for the L132C/T159S and L132A/T159C mutants. We also compared the deactivation kinetics (off rate) by fitting the decay time course of the currents evoked by a 10-ms light pulse at ND = 0 (Fig. 5C). Additionally, these mutants exhibited an increase in the off rate. Again, the increase in the off rate was particularly marked for the L132C/T159S and L132A/T159C mutants (Fig. 5D and Table 1).

To quantitatively examine the relationship between the increased operational light sensitivity and channel kinetics, the current amplitudes evoked by light at a ND = 2.5 (Fig. 5E) were plotted against the deactivation time constants (from Fig. 5D). The amplitudes of the evoked currents using this light intensity for these ChR2 mutants and wt-ChR2 were linearly correlated with the deactivation time constants (Fig. 5F).

In addition, two of the double mutants, L132A/T159A and 132S/T159A, exhibited extremely slow deactivation kinetics or off rates (Table 1). A long-lasting, sustained current could be evoked by a 10-ms light pulse, and deactivation could be accelerated by a pulse of long wavelength light (data not shown). The properties of these two mutants appear to be similar to the bi-step function of ChR2 mutants previously reported at the C128 site [25].

### Comparison of the Light Sensitivity of Mutant ChR2-expressing RGCs

**Figure 6:**
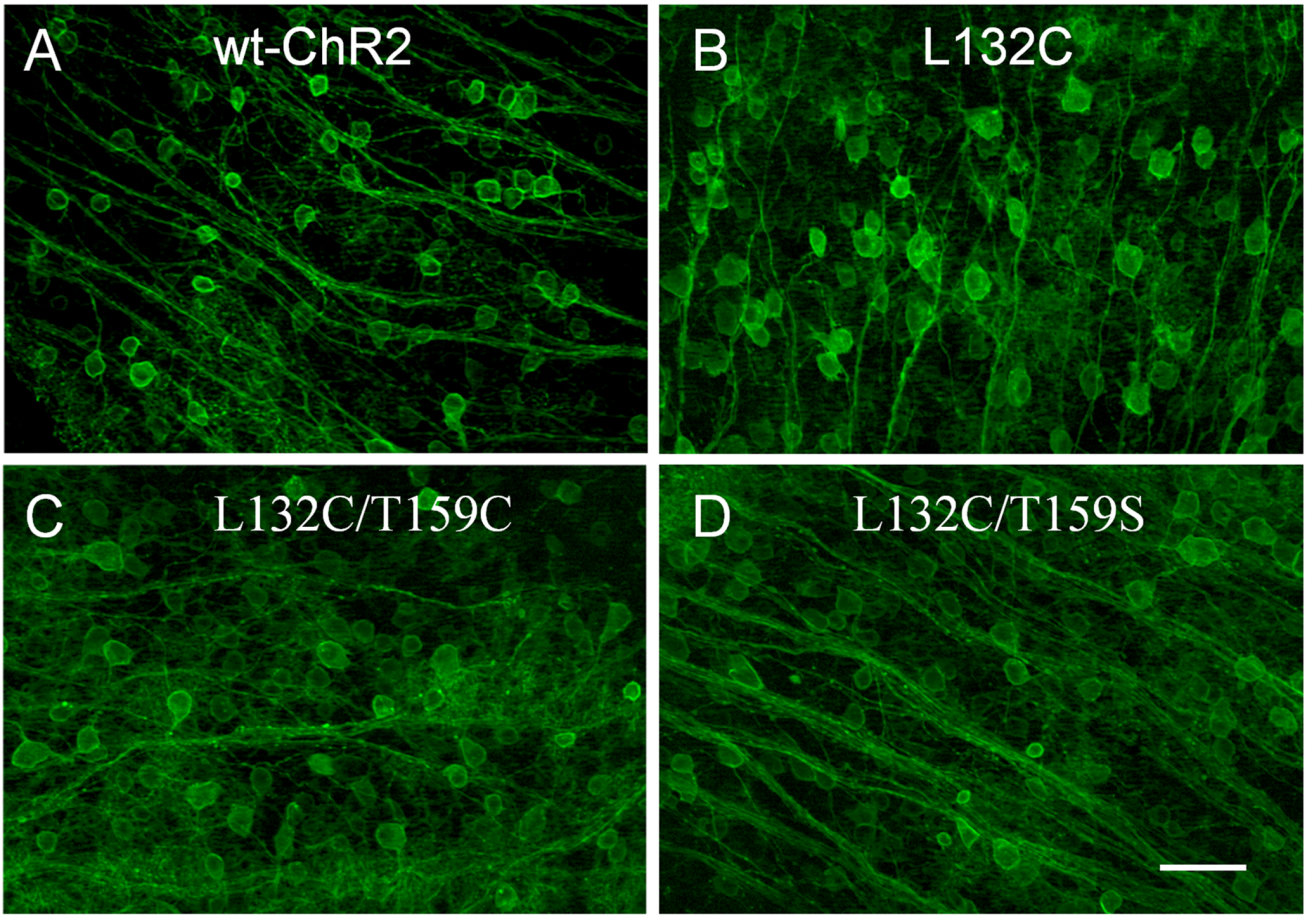
Representative fluorescence images of wt-ChR2 and three mutants show ChR2-GFP expression in retinal whole-mounts. All images were captured with the same exposure time and are displayed as maximum intensity projections. Scale bar, 50 µm.

Next, we examined the light response properties for some of the more light responsive mutants in RGCs. Thus, rAAV2 vectors were produced for the L132C, L132C/T159C, and L132C/T159S mutants (along with wt-ChR2). The viral vectors were injected into the eyes of mice through intravitreal administration. Similar to wt-ChR2 [1], all three of these ChR2 mutants displayed robust expression in cells located in the retinal ganglion cell layer, as viewed in retinal whole-mounts (Fig. 6).

**Figure 7:**
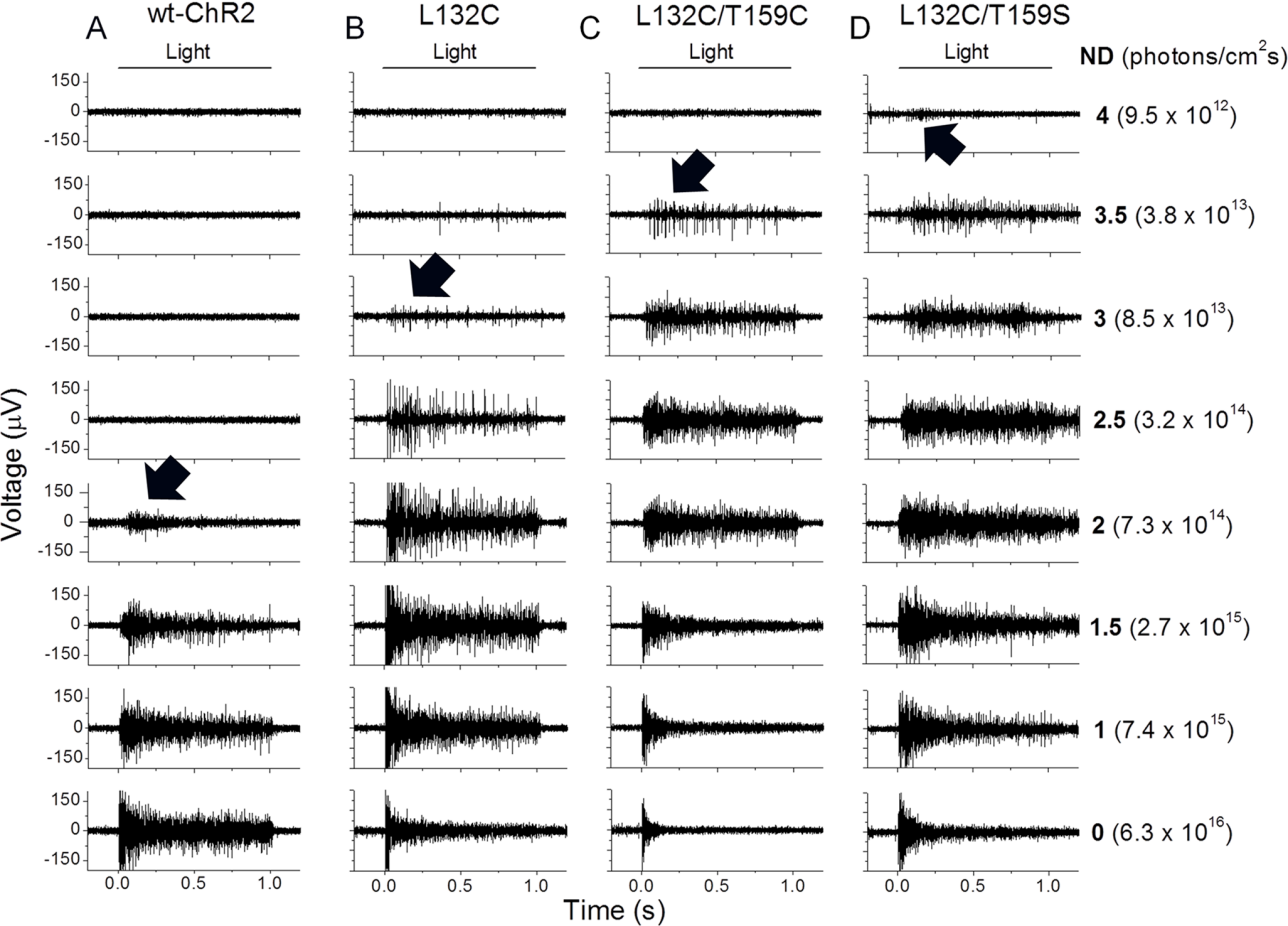
Multielectrode array recordings of RGCs from retinal whole-mounts to assess the threshold light intensities of the ChR2-mediated spiking activity. A–D) Representative recordings for ChR2-mediated spiking activity for wt-ChR2 (A), L132C (B), L132C/T159C (C), and L132C/T159S (D). The spiking activities were elicited by 1-s light pulses at different light intensities adjusted with neutral densities (ND) of 0, 1.0, 1.5, 2.0, 2.5, 3.0, 3.5, and 4.0. The threshold light intensities that were required to elicit spiking activity for wt-ChR2, L132C, L132C/T159C, and L132C/T159S were observed at NDs of 2.0, 3.0, 3.5, and close to 4.0, respectively (marked by arrows). The light intensities were measured as photons/cm^2^s.

The light sensitivity of RGCs from the retinas expressing wt and mutant ChR2s were compared by multielectrode array recordings. The photoreceptor-mediated light responses were blocked by CNQX (25 µM), APV (25 µM), and APB (10 µM). ChR2-mediated spiking activities were elicited by 1-s light pulses at different light intensities adjusted with neutral density (ND = 0 to 4.0) filters. Representative recordings are shown in Fig. 7. For wt-ChR2, the lowest light intensity required to produce detectable spiking activity, referred to as the threshold light intensity, was observed at approximately 2.0-ND attenuation. For the L132C and L132C/T159C mutants, the threshold light intensity was approximately 3.0- and 3.5-ND attenuation, respectively. For the L132C/T159S mutant, the threshold intensity was approximately 4.0-ND attenuation. The latter is nearly 2.0 log units lower than the threshold intensity of wt-ChR2.

Two additional properties were also observed. First, the spiking activity elicited from the L132C, L132C/T159C, and L132C/T159S mutants by high intensity light, ND≤1.5, exhibited apparent inactivation. This inactivation was more marked for the L132C/T159C mutant and was likely due to the inactivation of voltage-gated Na^+^ channels, which was caused by the large, sustained ChR2 current and thus the sustained membrane depolarization of RGCs. Second, for the L132C/T159S mutant, the spiking activity was prolonged after the termination of light stimulation. This result would be expected because of the slow deactivation kinetics of this mutant.

### Comparison of the Temporal Dynamics of Mutant ChR2-expressing RGCs

**Figure 8:**
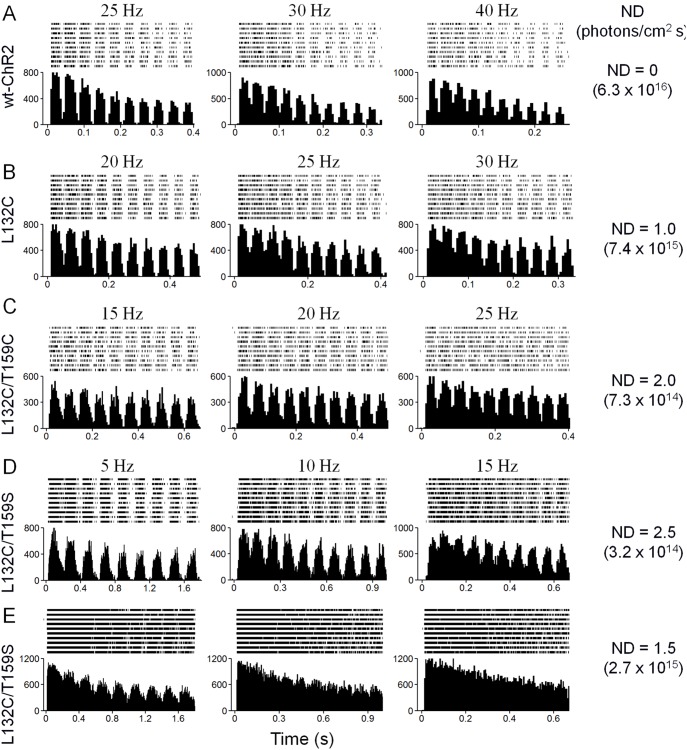
Multielectrode array recordings to assess the ChR2-mediated spiking activity of RGCs in response to flickering light stimuli. A–D) Representative recordings for ChR2-mediated spiking activity for wt-ChR2 (A), L132C (B), L132C/T159C (C), and L132C/T159S (D). In each panel, a raster plot of 10 recordings and an averaged spike rate histogram are shown in the top and bottom panels, respectively. The light intensities used were 0 and 1 ND attenuation for wt-ChR2 and L132C, respectively, and 2 and 2.5 ND attenuation for L32C/T159C and L132C/T159S, respectively. E) The responses from the same L132C/T159S mutant RGC to a higher light intensity with the same frequencies. The ability of the L132C/T159S mutant to follow the flickering light stimuli at all frequencies was deteriorated. The light intensities were measured as photons/cm^2^s.

We next examined how the slower kinetics of these mutants affected the temporal coding ability of RGCs in response to light stimulation. For this purpose, the spiking activity of RGCs expressing wt and mutant ChR2s was examined in response to different frequencies of flickering stimuli. Representative recordings from wt-ChR2 and the L132C, L132C/T159C, and L132C/T159S mutants are shown in Fig. 8A–D with light intensities for wt-ChR2 and the L132C mutant were approximately 2 log units above their threshold intensities and light intensities for the L132C/T159C and L132C/T159S mutants were approximately 1.5 log units above their threshold levels. The above-threshold intensities were chosen to demonstrate that sufficient light operation dynamics were retained for these mutants because the temporal coding ability was found to be worse at higher light intensities. As shown in Fig. 8E for the L132C/T159S mutant, the ability to follow the flickering light stimuli for the same RGC (Fig. 8D) to a higher light intensity (ND = 1.5) was markedly deteriorated at all frequencies.

**Figure 9:**
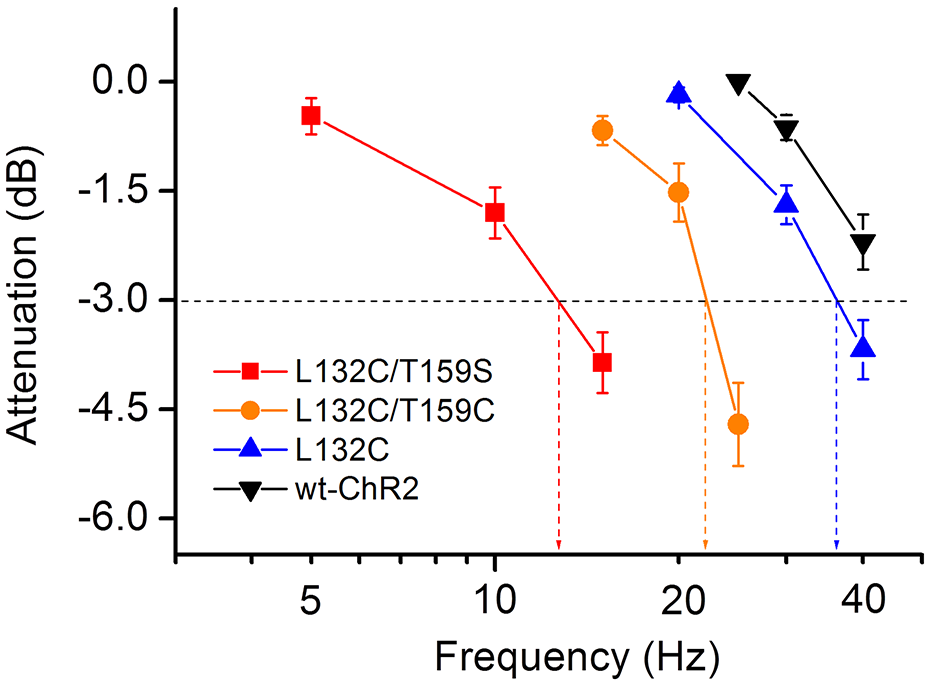
The relationship between response-amplitude-attenuation and frequency. The response-amplitude-attenuation at different frequencies for wt-ChR2, L132C, L132C/T159C, and L132C/T159S were measured. The attenuation values were converted to a decibel (dB) scale. The data are shown as the mean **±** SEM (*n* = 10 to 14). The horizontal dashed line indicates the −3 dB attenuation. The cutoff frequencies are estimated by the vertical dashed lines.

To provide a quantitative measure, the relationships between the response-amplitude attenuation and the frequency for the recordings under the conditions described in Fig. 8A–D are shown in Fig. 9. For wt-ChR2 and the L132C mutant, the cutoff or corner frequencies, defined as an attenuation of −3 dB, were at least 40 and 30 Hz, respectively. For the L132C/T159C and L132C/T159S mutants, the cutoff frequencies were at least 20 and 10 Hz, respectively.

## Discussion

### Mutations at L132 and T159 Affect ChR2 Expression, Operational Light Sensitivity, and Response Kinetics

Previous studies have reported the increase in photocurrent amplitude of ChR2 mutants at L132 and T159 [20], [21], [23]. In this study, we examined a number of single and double mutations at these two sites. We identified three additional single or double mutants, T159S, L132C/T159S, and L132A/T159C, that showed good or relatively good expression. We compared these three mutants with the previously reported mutants at these two sites. First, we found that a mutation at these two sites could markedly affect ChR2 expression as assessed by GFP expression in HEK cells. In fact, the majority of the examined mutants were found to have various expression problems. For many of these mutants, the expression of ChR2-GFP accumulated inside the intracellular organelles. Additionally, some mutants completely lost GFP expression. These results suggest that conformational changes caused by mutations at these two sites may affect ChR2 protein folding and/or intracellular trafficking.

Second, we found that mutations at these two sites could markedly alter the kinetics of ChR2-mediated currents, especially the deactivation kinetics. In fact, all of the mutants, including the previously reported L132C, T159C, and L132C/T159C mutants, showed slower deactivation kinetics compared with the kinetics of wt-ChR2. The off rate for wt-ChR2 was <20 ms. The off rate values for the mutants ranged from 45 ms for T159C to ∼1.4 s for L132A/T159C. The two mutants with the slowest deactivation constants, L132A/T159A and L132S/T159A, functioned as a bi-step-switch, similar to the ChR2 mutation previously reported at C128 [25]. In addition, the inactivation of ChR2 current was largely abolished in all of these mutants, except T159S, and in the previously reported T159C [20]. The T159S mutant was similar to the previously reported T159C mutant, but it had a slightly increased peak current and prolonged off rate.

Furthermore, all of the mutants exhibited an increase in operational light sensitivity, as evidenced by the increased current amplitude evoked by light with 2.5-ND attenuation. The operational light sensitivity should be dependent on the expression level, channel open probability, and single channel conductance [15]. Our results suggest that the expression levels among the six examined mutants were not markedly different, with the exception of the L132A/T159C mutant. The increase in operational light sensitivity was closely correlated with an increase in the off rate, again with the exception of the L132A/T159C mutant. The small deviation to this correlation for the L132A/T159C mutant is likely due to its expression problem. Therefore, our results suggest that the increase in operational light sensitivity for these mutants could be largely explained by an increase in the channel open probability, as reflected by a prolonged off rate.

### Relationship of Light Sensitivity and Temporal Dynamics of ChR2 Expressing RGCs

In this study, we compared the light sensitivity and temporal dynamics in mouse RGCs expressing three mutants, L132C, L132C/T159C, L132C/T159S, and wt-ChR2. The light sensitivity of these mutant-expressing RGCs was increased compared with the sensitivity of wt-ChR2. This difference in sensitivity was demonstrated by directly examining the threshold light intensity that was required to produce ChR2-mediated spiking activity. The light sensitivity for the L132C, L132C/T159C, and L132C/T159S mutants was increased by approximately 1, 1.5, and close to 2 log units, respectively, compared with wt-ChR2. Thus, the L132C/T159S mutant-expressing RGCs appeared to be the most light-sensitive.

Of note, the light intensities that were required to evoke threshold spiking activity in RGCs were much lower than the intensities expected from the HEK cell recordings. For example, for wt-ChR2, the threshold light intensity that evoked spiking activity was 7.3×10^14^ photons/cm^2^s (Fig. 7A). In comparison, this light intensity does not appear produce a noticeable current in HEK cells (Fig. 4). However, the average current evoked from wt-ChR2 in HEK cells by light at 1.4×10^15^ photons/cm^2^s was 6.5 pA (Fig. 4A and B). This current is sizable for RGCs. Moreover, considering the large dendritic trees of RGCs, the elicited current in RGCs by the same light intensity could be much larger. Thus, it would not be unexpected for this or a slightly lower light intensity (e.g., 7.3×10^14^ photons/cm^2^s) to elicit spiking activity in RGCs. Nevertheless, this phenomenon does emphasize an important fact; the absolute current amplitude elicited at a low light intensity is essential in determining the functional sensitivity of ChR2 mutants in RGCs.

Although the ChR2-mediated current evoked by low light at ND = 2.5 for the L123C/T159S mutant was more than double compared with the L132C/T159C mutant, the threshold light sensitivity in RGCs was increased by less than a half log unit. This less than expected increase in light sensitivity for the L132C/T159C mutant-expressing RGCs is likely due to the marked decrease in current activation kinetics or on rate for this mutant (Table 1) because the ability to elicit neuron spiking would be determined not only the magnitude of the current but also by the speed of the current activation.

Furthermore, our results showed that the decreased off rate affected the temporal signal code ability in RGCs. For all mutants, a higher operational light sensitivity was correlated with a decreased off rate, which lowers the maximal flickering light frequency that the RGCs can follow. Nevertheless, the ChR2-mediated spiking activity for the RGCs that expressed the two most light responsive mutants, L132C/T159C and L132C/T159S, could still follow flickering light stimuli up to 20 and 10 Hz, respectively. Because the cutoff frequencies for these two mutants were obtained at light intensities approximately 1.5 log units above their threshold light intensities, our results indicate that these mutants also retain reasonably good light operation dynamics.

### Implications and Further Development of Optogenetic Sensors for Restoring Vision

The results of this study are consistent with previous reports that ChR2 mutations at L132 and T159 can markedly increase operational light sensitivity because of an increase in the magnitude of the ChR2 current evoked with low light. In addition, we identified additional mutants with further increases in operational light sensitivity. An important conclusion revealed in this study is that the increased operational light sensitivity for all of the mutants was always correlated with decreased current kinetics, both activation and deactivation. This also appears to be a general property for all ChR variants that have been reported to date [15]. The decrease in activation kinetics will eventually limit the maximal threshold light sensitivity of RGCs despite the increase in ChR-mediated current to low light. The decreased deactivation kinetics will affect the temporal coding ability of RGCs to light signals.

However, unlike many other optogenetic applications that require fast light sensors [19], [26], [27], fast temporal ChR kinetics are not essential for the purpose of vision restoration because the human eye is most sensitive to stimuli modulated at temporal frequencies of 15 to 20 Hz at high luminance, with a critical flicker frequency of 15 Hz for scotopic vision [28]. Thus, the use of ChR for visual prosthesis could tolerate slower kinetics with high operational light sensitivity. ChR2 mutants, such as L132C/T159C and L132C/T159S, that can follow flickering light up to 20–10 Hz may be suitable for the application of vision restoration. Although the further development of ChR2 or ChRs with higher operational light sensitivity but fast kinetics is still needed, and especially with the discovery of these highly light responsive ChR2 mutants, the low light-sensitivity of ChR-expressing cells may no longer be a major hurdle for vision restoration. Furthermore, targeted expression of these highly light responsive ChR2 mutants to more distal inner retinal neurons, such as retinal bipolar cells [5], [8] or AII amacrine cells [29], could further reduce the required light intensities because of the signal convergence from these cells to RGCs [30], [31]. The light sensitivity of ChR2 or ChRs could also be partially compensated for with an extra ocular imaging device. An extra ocular imaging device is likely to be required to expand the operational capabilities of the optogenetic-based retinal prosthesis.

To further improve the properties of ChR2 or ChR for vision restoration, one possible solution is to create ChR2 mutants or ChR variants with optimized off rates, possibly between 200 and 1000 ms. Such mutants may achieve the highest operational light sensitivity in retinal neurons with an optimum balance between the current magnitude and activation kinetics. This type of mutant could also result in faster temporal kinetics than the kinetics of the L132C/T159S mutant. Ideally, to increase the light sensitivity of ChR-expressing neurons without sacrificing the kinetics would require creating ChR mutations that would increase channel conductance; however, no such ChR mutant has been reported. In addition, the use of red-shifted ChRs [17], [22], [23], [32] or the creation of ChRs with a broad spectrum could further improve the light sensitivity of ChR-expressing inner retinal neurons to natural daylight and thus their suitability vision restoration applications.
